# Clinical Report of Cases Treated with Phylacogen*Read before The County Medical Society, Fort Madison, Ia.

**Published:** 1913-02

**Authors:** F. C. Smith

**Affiliations:** Keokuk, Ill.; No. 310 Main Street


### Clinical Report of Cases Treated With Phylacogen*

By F. C. SMITH, M.D.
Keokuk, Ill.

Having been invited to present to the members of this Society a report, embodying my experiences with Phylacogen, it gives me pleasure to offer the following report:

The following is my experience with five cases:

One acute muscular rheumatism, cured.

One gonorrheal arthritis, cured.

One hay fever, not benefitted.

One arthritis, not benefitted.

One gonorrheal orchitis, cured.

These cases briefly are as follows:

*Case No. 1.*—Acute Inflammatory Rheumatism, J. F. P., age 38, male, married; occupation, traveling salesman. Has suffered from repeated attacks of acute inflammatory rheumatism, complained of attacks occurring in one joint and then in another, not confined to any one locality. In each instance attended by swelling, pain and fever, typical symptoms of acute inflammatory rheumatism. The treatment with Rheumatism Phylacogen was first instituted April 12th, 1912, with 5 c. c., given under the skin. This dose repeated on the next day. On the third day 10 Cc. were given, followed by 10 Cc. on each of the three succeeding days. Six injections were given, no decided reaction was noticeable. The temperature was generally around 100 and a fraction before injection. I did not see the patient for two hours and a half after the sixth injection, at that time he had passed reaction, his temperature was 102 3/5, patient had a very profuse perspiration and seemed prostrated. The family were much frightened at the reaction and from what they stated it appeared that he had a heavy chill with high fever and was somewhat cyanosed. Patient had been confined to bed for some time, but the next day, after the sixth injection, was up and around the house. He refused further injections, claiming that he was completely relieved.

*Case No. 2.*—Patient L., age 28, male, married; occupation, locomotive fireman. This patient previously had an attack of acute specific urethritis which was later followed by the involvement of the knee joints which became much swollen and so painful that he was unable to use them, motion being very painful, his temperature ranged from 100 to 102, his knees were badly swollen and he was confined to bed, diagnosis was Gonorrheal Arthritis. On July 6th, 1912, Gonorrheal Phylacogen was administered in a dose of 10 Cc., repeated every day until July

* Read before The County Medical Society, Fort Madison, Ia.

17th, a total of 120 Cc. being given, and then every second day until 70 Cc. additional had been administered. This patient then received in all 190 Cc. during twenty-five days, at which time he was discharged with complete relief of all symptoms.

*Case No. 3.*—Patient A. A., age 28, male, married; occupation, laborer. Gave history of a previous attack of specific urethritis with involvement of the left testicle. When the patient presented himself this organ was swollen, tense and very painful. With the previous history and the symptomatology it was not difficult to make a diagnosis of Gonorrheal Arthritis or Epididymitis. Gonorrhea Phylacogen was administered in 3Cc. doses night and morning for two days, increased on the third day to 5 Cc., night and morning. For the first three days there was some rise in temperature, the highest point being 102¾. Continued the treatment for four days longer with no more reaction. The swelling of the testicle subsided rapidly and this condition was completely cured. The acute urethritis, however, persisted.

*Case No. 4.*—Patient R. R., age 44, male, married; occupation, clergyman. Gave a history of having hay fever every year. On July 23rd presented himself with the typical symptoms of hay fever, at which time 5Cc. of Mixed Infection Phylacogen were given, followed by 10 Cc. on July 24th. There was some reaction following these two injections, but no benefit. The patient received no further treatment and the case was not cured. It should be understood that "One swallow does not make a Summer," and it should likewise be understood that no distinct claims have been made as to the definite cure of hay fever with Phylacogen. One naturally hesitates to discuss hay fever with a biological product like Phylacogen because of the ideas which have prevailed in the profession concerning the cause of hay fever. I am informed that a number of physicians throughout the United States have reported a large number of cases of hay fever as symptomatically cured by the use of Mixed Infection Phylacogen. At first these cases were being treated with 5 and 10 Cc. doses; the reaction, however, alarmed some of the patients with the result that the experiment of giving small doses was instituted, and this has resulted in diminishing the reaction quite decidedly and enabling the patients to persist with the treatment for a sufficient length of time, the net result has been that a very large majority of the cases have been relieved, so it is reported.

*Case No. 5.*—Patient A. A., age 29, male, married; occupation, Cabinetmaker. Four years ago patient noticed a swelling in the popliteal spaces which gradually increased and interfered with flexion of the leg. When the patient consulted me there was a large swelling of both knee joints, considerable pain on motion, the joints were hot and tender, the patient was unable to walk without crutches, the patient denied any specific infection. A provisional diagnosis was made of arthritis and Rheumatism Phylacogen was administered, beginning on July 6th and continuing every day until 170 Cc. had been given, followed by no reaction, except on July 15th, when the temperature rose to 100 2/5, when the treatment was discontinued.

In addition to the above I will give a summary of cases not included in my original report.

Fourteen cases as follows:

Seven cases Orchitis and Epididymitis.

Five cases cured.

One case benefitted.

One case not benefitted.

Seven cases Rheumatism.

Four cases cured.

One case benefitted.

Two cases not benefitted.

This necessarily brief clinical experience is related for the purpose of stating what I have personally observed in the treatment of certain conditions with this new remedical agent. Phylacogens are not bacterial vaccines or antitoxins, as we ordinarily understand these terms. They are somewhat similar to bacterial vaccines, in that they are sterile aqueous solutions of certain, at present unknown, metabolic substance or derivatives generated by bacteria when grown in artificial media. The germs are killed and then removed by filtration through porcelain, the filtrate is sterile and to insure this Phylacogen is subjected to careful cultural tests.

The Phylacogens are made from a large variety of pathogenic bacteria, such as the several staphylococci, Streptococcus pyogenes, Bacillus pyocyaneus, Diplococcus pneumoniae, Bacillus typhosus, Bacillus coli communis, Streptococcus rheumaticus, Streptococcus erysipelatis, etc. This basic Phylacogen is a "polyvalent" preparation, or Polyphylacogen since the organisms are not from one strain only of a given species, but from cultures made at frequent intervals and from a variety of sources. The various organisms are present in the material before filtration and dilution in approximately equal proportions. The cultures are incubated at 37° C. for 72 hours or longer, after which a preservative consisting of 0.5 per cent. of phenol is added to the fluid, which is then filtered through porcelain. The basic Phylacogen, made in this manner, is used in the preparation of the several specific Phylacogens. It has been named "Mixed Infection Phylacogen."

Each specific Phylacogen is prepared as follows: The basic material (Mixed Infection Phylacogen) is modified by the addition of an equal amount of the filtrate obtained by growing and treating the organism considered to be predominant in the pathological condition to be treated, for instance, in the preparation of Gonorrhea Phylacogen, the gonococcus is grown and treated like the several organisms entering into the preparation of the basic Mixed Infection Phylacogen. The filtrate obtained from the preparation of the gonococcus organism alone is added in equal amount to the Mixed Infection Phylacogen, and the resulting product given the specific name "Gonorrhea Phylacogen." A similar method is employed in the manufacture of the other specific Phylacogens, such as Pneumonia, Erysipelas, etc., etc.

Phylacogens were originated by Dr. A. F. Schafer of Bakersfield, Cal. Dr. Schafer presented his method of preparing Phylacogens and his technique of their application to the San Joaquin Medical Society in Fresno, California, in October, 1910, and later to the San Francisco Medical Society, January 14, 1911, and published a preliminary statement in the Therapeutic Gazette for April, 1911.

Since the appearance of Dr. Schafer's article a number of physicians have reported their experiences, entitled the following:

No. 1. "Results Obtained With Modified Vaccines," by L. D. Green, M. D., San Francisco, Cal.; published "Cal. State Jour. of Med.," April 12th, 1912.

No. 2. "On Gonorrhea Phylacogen," by G. K. Swinburne, M. D., New York, N. Y.; published "New York Med. Record," May 25th, 1912, page 975.

No. 3. "Schafer's Treatment for Rheumatism," by G. C. Crandall, M. D., St. Louis, Mo.; published "Mo. State Med. Jour.," June, 1912, page 492.

A large number of other papers have also been printed, the consensus of opinion being that Phylacogens mark a new era in the treatment of infections and also that many more conditions are due to infection than have previously been suspected or proved.

### THEORY: THE VIEWS OF DR. SCHAFER.

The principle upon which the use of these Phylacogens is founded is, briefly, the theory of multiple infections. This principle is supported by an extraordinary practical experience, supplemented by exhaustive and long-continued clinical experimental work by Dr. Schafer.

Three facts are set forth by Dr. Schafer as the basis of this new therapy.

First: Practically all acute and many of the chronic diseases are caused by the metabolic products of bacteria.

Second: The human subject is the host of micro-organisms that are pathologically latent but capable of setting up a disease process under certain conditions.

Third: The growth of infecting micro-organisms can be arrested and their effects neutralized by products derived from their development in artificial culture media.

Dr. Schafer is of the belief that all infections are "Mixed Infections;" that except in rare instances there is no such thing as an infection by a single species of micro-organisms; that while one species may predominate, the pathologic process engendered by it is accelerated and intensified by the presence of organisms of other species; in other words, that in the course of an infectious disease the symptoms are due not only to the effects of a single species of organisms (the specific infection), but to the influence of other organisms whose pathogenic role is not insignificant, but which must be reckoned with in any successful scheme of therapeutics.

Dr. Schafer further believes that the human subject is at all times the host of a great variety of organisms and may harbor pathogenic bacteria without harm to itself during periods of physiologic resistance at or above par and in the absence of any solution of tissue continuity. When the resistance is below par, or a solution of continuity of tissue occurs, the bacteria harbored by the human host assume pathologic significance.

Furthermore, he contends that certain diseases, as typhoid fever, pneumonia, tuberculosis, erysipelas, rheumatism and others, are objective and subjective symptomatic manifestations of the preponderance in the patient of the toxic and destructive products of the peculiar species of organism to which the etiology of the disease is usually ascribed as B. typhousus in typhoid fever, D. pneumoniae in pneumonia, B. tuberculosis in tuberculosis, etc., and, in addition, the symptoms are due in part at least to the destructive action of certain materials produced by complicating organisms which are always present in great variety and number.

As an illustration, attention may be directed to the now commonly accepted idea that in pulmonary tuberculosis the greatest danger to the patient, much of the difficulty of the treatment, and many of the most notable symptoms, such as loss of weight, high temperature, disturbance of circulation, purulent expectoration, destruction of tissue, etc., are due to the complicating organisms, and if the so-called "mixed infection" can be checked or eliminated, efforts may be directed against the bacillus tuberculosis with far greater success than has heretofore been possible in the treatment of this condition.

Dr. Schafer points to the fact that the administration of bacterial vaccines to patients suffering from infection not infrequently fails of effect because the truth of the above assumption is not recognized, especially when the treatment, being based upon the opsonic theory, consists in the use of a vaccine made from a single species of organisms isolated from the patient. Bacterial vaccines made from a single species of organism prove successful in many cases, but the multiplicity of "combined" bacterial vaccines now in use points to the rapidly developing conclusion that the great majority of patients require something more than treatment with a vaccine made from one organism; the success attending the use of bacterial vaccines made from a number of different species, even when used in cases apparently due to one species, points to the likelihood of this theory being correct.

While my own personal experiences have not been very extensive, embracing so far 19 cases, as reported, with 14 successful results and 5 failures to obtain benefit; the successes are sufficiently definite and striking to warrant the application of Phylacogen wherever indicated.

No. 310 Main Street.

---

Feeble Minded Heredity.—Miss McKinnie, *Public Health* (Bulletin of Michigan Board of Health), October, 1912. The study is of the descendents of a brother and sister, the latter feeble minded, who married normal individuals. The sister had 12 children, including a still birth. Among these, four were normal and the state of one was not known. Of the other six, three were feeble minded, one alcoholic, one epileptic and one was a sexual pervert (female). The (normal) brother had one normal son who married a feeble minded woman. They had seven children, 5 of whom died in infancy or childhood. The remaining two were feeble minded girls. One of these married her feeble minded second cousin of the older generation mentioned above. From this last union, sprung 11 children. Six of these died in infancy. Of the remaining 5, three were distinctly feeble minded, one unknown and one doubtfully normal. (Note—While the high mortality in such marriages is conservative, it is questionable how much of it is due to instrinsic lack of vitality and how much to privation and neglect. For example, one feeble minded couple known to the editor, used to buy a bag of flour, make it up into dough in a tub, at once, and use the sour dough, almost to the exclusion of other food, as long as it lasted. Quite aside from the ultimate object of eugenics, think how much suffering would be saved from a few properly timed oophorectomies and ligations of the vas deferens.)

